# The Druggist’s General Receipt Book

**Published:** 1853-05

**Authors:** 


*The Druggist's General Receipt Book.* By Henry Beasley, second American from the last London edition, corrected and enlarged. Philadelphia: Lindsay & Blackiston, 1852.

THIS book contains receipts for medicines for horses, for cattle, for sheep or lambs, for swine, for dogs, for poultry, for rabits, patent and proprietary medicines, Druggist's nostrums, perfumery, cosmetics, beverages, dietetic articles and condiments, trade chemicals, &c. We are sorry the publishers have made it mechanically so good a book; for their sake we advise all quack doctors to purchase it. J.

